# Genome-Wide Association of Copy Number Polymorphisms and Kidney Function

**DOI:** 10.1371/journal.pone.0170815

**Published:** 2017-01-30

**Authors:** Man Li, Jacob Carey, Stephen Cristiano, Katalin Susztak, Josef Coresh, Eric Boerwinkle, Wen Hong L. Kao, Terri H. Beaty, Anna Köttgen, Robert B. Scharpf

**Affiliations:** 1 Department of Epidemiology, Johns Hopkins Bloomberg School of Public Health, Baltimore, Maryland, United States of America; 2 Division of Nephrology and Hypertension, Department of Internal Medicine, University of Utah School of Medicine, Salt Lake City, Utah, United States of America; 3 Department of Biostatistics, Johns Hopkins Bloomberg School of Public Health, Baltimore, Maryland, United States of America; 4 Renal Electrolyte and Hypertension Division, Perelman School of Medicine, University of Pennsylvania, Philadelphia, Pennsylvania, United States of America; 5 Welch Center for Prevention, Epidemiology and Clinical Research, Baltimore, Maryland, United States of America; 6 Human Genetics Center, University of Texas Health Science Center at Houston, Houston, Texas, United States of America; 7 Division of Genetic Epidemiology, Medical Center–University of Freiburg, Faculty of Medicine, Freiburg, Germany; 8 Department of Oncology, Johns Hopkins School of Medicine, Baltimore, Maryland, United States of America; Istituto Di Ricerche Farmacologiche Mario Negri, ITALY

## Abstract

Genome-wide association studies (GWAS) using single nucleotide polymorphisms (SNPs) have identified more than 50 loci associated with estimated glomerular filtration rate (eGFR), a measure of kidney function. However, significant SNPs account for a small proportion of eGFR variability. Other forms of genetic variation have not been comprehensively evaluated for association with eGFR. In this study, we assess whether changes in germline DNA copy number are associated with GFR estimated from serum creatinine, eGFRcrea. We used hidden Markov models (HMMs) to identify copy number polymorphic regions (CNPs) from high-throughput SNP arrays for 2,514 African (AA) and 8,645 European ancestry (EA) participants in the Atherosclerosis Risk in Communities (ARIC) study. Separately for the EA and AA cohorts, we used Bayesian Gaussian mixture models to estimate copy number at regions identified by the HMM or previously reported in the HapMap Project. We identified 312 and 464 autosomal CNPs among individuals of EA and AA, respectively. Multivariate models adjusted for SNP-derived covariates of population structure identified one CNP in the EA cohort near genome-wide statistical significance (Bonferroni-adjusted p = 0.067) located on chromosome 5 (876–880kb). Overall, our findings suggest a limited role of CNPs in explaining eGFR variability.

## Introduction

Chronic kidney disease (CKD) is defined by reduced kidney function or kidney damage and can progress over time. It is estimated that CKD effects about 26 million US adults[1] and its prevalence is increasing both in the United States and globally[1, 2]. CKD has a genetic component, which may contribute to the development or progression of CKD in addition to or through established epidemiological CKD risk factors such as hypertension, diabetes, and proteinuria. Genome-wide association studies limited to single nucleotide polymorphisms (SNPs) have identified more than 50 loci associated with estimated glomerular filtration rate (eGFR), a measure of kidney function, in European ancestry (EA) populations[3–5]. These loci account for only 3% of the variation in eGFR[3], while the heritability of eGFR has been estimated at 33%–75% [6–9]. We hypothesized that heritable loss and gain of germline DNA copy number may contribute to kidney function.

Glomerular filtration rate (GFR) is considered the best quantitative measure of kidney function. However, gold standard estimates of GFR by urinary or plasma clearance of exogenous filtration markers are too cumbersome and expensive for use in clinical and research settings. Serum creatinine has emerged as a reliable indicator of GFR[10] and is currently the most commonly used biomarker of kidney function.

Copy number variants (CNVs) have been reported to encompass genes involved in the regulation of cell growth and metabolism, implicating vital roles in the variability of human traits and disease risk[11–13]. Copy number polymorphisms (CNPs), regions of the genome where CNVs segregate in the germline at a population frequency of at least 2 percent, have been implicated in a broad range of human diseases, including mental health (bipolar disease[14], schizophrenia[15–17], and autism spectrum disorder[18, 19]), metabolic disease (type I diabetes[20] and obesity[21–23]), congenital anomalies (kidney and urinary tract defects[24–26], oral clefts[27–29]), and cancer (breast cancer[30], melanoma[31], and colorectal cancer[32]). While previous SNP association studies have identified several loci strongly associated with kidney disease, the causal variants are generally not known. CNPs occurring at known risk loci may help establish a genetic basis for this disease. For example, a deletion of any part of a gene or its promoter can disrupt transcription to mRNA; amplification of a gene can lead to over expression of the mRNA product. In Scharpf *et al*., 2014[33], we showed the association between copy number and uric acid levels at *SLC2A9* was independent of nearby SNP-association signals. As many array platforms include probes targeting regions of the genome that are monomorphic at the single nucleotide level (i.e. there is only one allele at the probe), the discovery of risk loci not well tagged by SNPs is also possible. The array platform used in ARIC has approximately 1 million monomorphic probes in addition to 1 million polymorphic (SNP) markers, enabling identification of CNPs in regions not well tagged by SNPs.

Here, we use Bayesian Gaussian Mixture Models (GMMs) to estimate copy number at polymorphic (> 2% of subjects) regions identified by a Hidden Markov Model (HMM) and regions previously reported as polymorphic in the HapMap Project[34]. We evaluated models for eGFR by serum creatinine (eGFRcrea) levels that include copy number at CNP regions as a covariate. Findings presented here are the most comprehensive analyses to date of CNPs and quantitative measures of kidney function in an adult population. Further, this is the first study to present genomic analyses of copy number for individuals of AA in the ARIC study.

## Results

The ARIC study includes 9,483 EA and 2,822 AA participants with both baseline eGFRcrea and Affymetrix 6.0 genotype data. The EA and AA cohorts differ in known clinical risk factors for kidney disease and eGFRcrea levels (Table 1). In particular, the percentage of AA participants with hypertension was 56.5, more than twice the percentage among EA participants (26.6 percent). Similarly, the percentage of AA with diabetes was 19.3 percent compared to only 8.6 percent in the EA cohort. The average eGFRcrea among AA participants was 103.2 ml/min/1.73m^2^, compared to an average of 89.8 ml/min/1.73m^2^ among EA subjects.

**Table 1:** Study sample characteristics. Descriptive statistics are shown as mean and (standard deviation) unless otherwise indicated.

	European ancestry	African ancestry
Sample size eGFRcrea/eGFRcys	8645/6843	2514/1673
Women, N (%)	4592 (53.1)	1576 (62.7)
Age (years)	54.2 (5.7)	53.5 (5.8)
Center N (%)	F 2606 (30.1)	F 288 (11.5)
	J 0 (0)	J 2226 (88.5)
	M 3226 (37.3)	M 0 (0)
	W 2813 (32.5)	W 0 (0)
eGFRcrea (ml/min/1.73m^2^)	89.8 (18.0)	103.2 (25.0)
eGFRcys (ml/min/1.73m^2^)	84.3 (19.6)	91.7 (24.9)
HTN, N (%)	2288 (26.6)	1413 (56.5)
DM, N (%)	745 (8.6)	484 (19.3)

**Fig 1:**
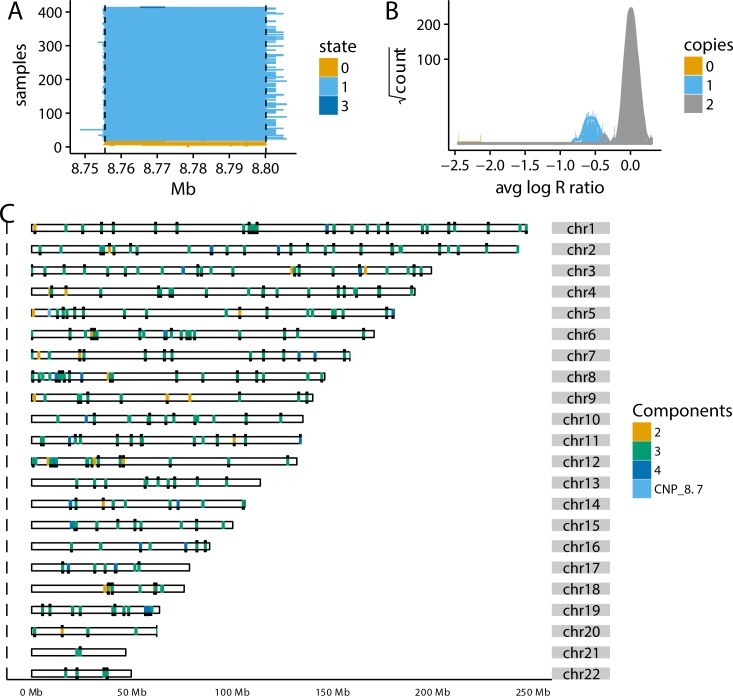
Developing a profile of autosomal CNP regions in ARIC. (A) CNVs identified from the HMM often have similar genomic endpoints across samples, shown here as colored rectangles for ~450 EA participants at a region on chromosome 5 (top signal in EA analysis). (B) The distribution of the average for 8,645 EA participants at the region on chromosome 5 approaching genome-wide significance. Copy number is called by the maximum *a posteriori* estimates from a normal mixture model. (C) All autosomal CNP regions identified either by HapMap or from the HMM among EA participants color-coded by the number of copy number states. Black ticks above the ideograms are additional regions from HapMap identified as polymorphic by the GMM in ARIC. Black ticks below the ideograms are CNPs that are also present in the AA cohort (see also Figure C in S1 File). The region on chromosome 5p is highlighted.

To identify CNVs, we fit a 6-state HMM for all participants passing previously established quality control steps[35]. Additional statistics for quality control in this study include the median absolute deviation (MAD) and lag-10 autocorrelation of autosomal log_2_ R ratios (LRRs) (Figures A and B in S1 File). On average, the HMM identifies more CNVs in participants of AA than EA with median frequencies of 68 and 57, respectively. Among EA participants, approximately 10% of the CNVs span fewer than 10 SNPs or monomorphic markers and were excluded from further analysis. Of the remaining CNVs, approximately 64% (429,162) occur at regions that are copy number altered in 2 percent or more of the EA or AA participants (e.g., Fig 1A). Hereafter, we refer to these regions as CNPs.

A major challenge for identifying CNVs is the substantial intra-subject variance of the LRRs. As we and others have demonstrated[34–36], the signal to noise ratio can be improved by modeling the distribution of only the markers involved in the polymorphism across many subjects. To comprehensively identify CNPs, including those too small to be estimated by the HMM, we evaluated 785 additional regions reported as polymorphic in HapMap[34] and spanning 3 or more Affymetrix 6.0 markers. For HapMap regions that overlap the HMM-derived CNPs, we used the genomic coordinates from the HMM.

For each candidate CNP, maximum *a posteriori* estimates of relative copy number were obtained from a GMM implemented in the R package cnvCall[37]. Excluding monomorphic regions, we identified 312 and 464 autosomal polymorphic regions in the EA and AA cohorts, respectively (Fig 1C and Figure C in S1 File). After translating mixture component indices to copy number and manually recalling rare homozygous deletions (see Methods), we found roughly 85 percent of the deletion CNPs in the EA and AA cohorts occur at frequencies consistent with Hardy Weinberg equilibrium (p > 0.01; Figure D in S1 File).

**Fig 2:**
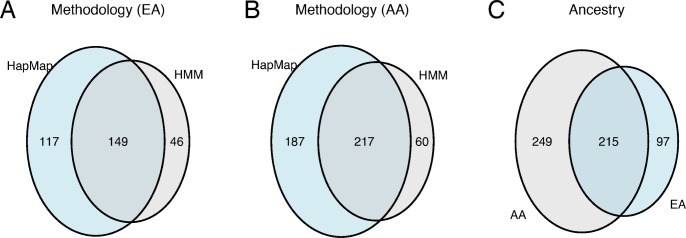
Overlap of CNP regions by methodology of identification and ancestry. (A, B) The number of CNPs identified by methodology for EA (left) and AA ancestry (middle). (C) The overlap of polymorphic regions by ancestry. The EA and AA cohorts shared 215 CNP regions.

To contrast the CNP regions by methodology (HapMap or HMM), we assessed the extent to which the CNP regions overlap. Interestingly, 15% of the EA CNPs (n = 46) and 13% of the AA CNPs (n = 60) did not overlap with published regions in HapMap (Fig 2A and 2B). To evaluate whether the CNPs identified by only the HMM (not reported in HapMap) were common in other studies, we examined 17 studies each having at least 100 subjects deposited in the Database of Genomic Variants as of May 15, 2016 (http://dgv.tcag.ca, NCBI build 36). With few exceptions, nearly all of these CNPs were in one or more of these studies at a frequency of 2 percent or more (Figure E in S1 File).

Comparing CNP coordinates by ancestry, we find that only 215 of the 464 regions identified in the AA cohort are also polymorphic in the EA cohort (Fig 2C). As the ratio of EA to AA participants in ARIC was more than 3:1, an unstratified analysis would have failed to identify regions absent among EA participants and occurring in less than 10 percent of AA participants.

**Fig 3:**
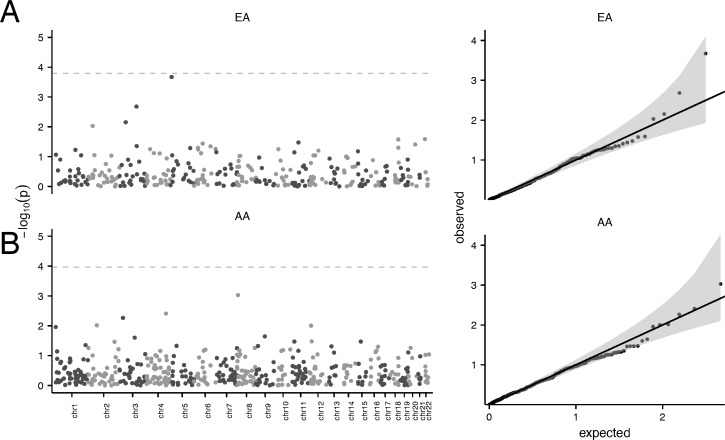
Statistical significance of copy number in linear regression models for eGFRcrea. (A) Manhattan plot for CNP association analysis in eGFRcrea among 8,645 European ancestry and 2,514 AA participants in the ARIC study. The gray line indicates genome-wide statistical significance. (B) Quantile-quantile plots of the expected–log 10 p-values under the null hypothesis of no association versus the observed–log 10 p-values. The lower and upper bounds of the shaded region indicate 0.025 and 0.975 quantiles, respectively, of the null.

To assess whether copy number at identified CNP regions is associated with eGFRcrea, we evaluated linear models with log-transformed eGFRcrea as the dependent variable with copy number and SNP-derived covariates of population structure as covariates (Methods). Because global differences in allele frequencies may exist between subpopulations particularly within the AA cohort, we estimated the percentage of African ancestry by ANCESTRYMAP[38] using genotypes from 1401 ancestry-informative markers in 2,152 AA participants. For the AA cohort, none of the AA CNPs are statistically significant with or without global ancestry adjustment (Fig 3A). For the EA cohort, none of the models are statistically significant after Bonferroni-correction (Fig 3B), though CNP_8.7 (chr5: 8,755,522–8,800,142 bp) is suggestive (adjusted p = 0.067).

CNP_8.7 is a deletion polymorphism located in a gene desert[39, 40] and is associated with a 0.04 increase in log eGFRcrea (95% CI: 0.02–0.06 ml/min/1.73 m^2^). The deletion allele is interrogated by 40 monomorphic markers and 7 SNPs on the Affymetrix platform and overlaps deletions previously identified in HapMap[34]. The deletion allele segregates in the EA population at Hardy Weinberg equilibrium (p = 0.4). In particular, 393 (4.5%) hemizygous deletions, 2 (0.02%) homozygous deletions, and 8,267 (95.6%) with diploid copy number were identified.

To confirm copy number estimates at CNP_8.7 with an alternative technology, we obtained next generation sequencing data from dbGaP for five subjects with a putative hemizygous deletion (accession number phs000090.v1.p1). Preprocessing the read depth in 10kb bins to adjust for GC-content and excluding regions of low mappability (see Methods), we find that all five samples have two or more 10kb bins in the region with log ratios of relative copy number less than -1 (Figure F in S1 File). Further, the three samples with lowest technical variation in the sequencing platform (F159225, F264060, and W156974) have approximately the same boundaries as identified by the array platform.

In the absence of any stronger candidate than CNP_8.7 to pursue for replication in an independent study, we evaluated an alternative GFR surrogate. In particular, we hypothesized bona fide modulation of latent GFR by copy number dosage would be captured by multiple GFR surrogates. As cystatin C is also available in ARIC and well regarded as a surrogate for calculating eGFR[41], we evaluated the same regression model as described previously with eGFR by cystatin C (eGFRcys) as the dependent variable. For the 6,830 subjects with available eGFRcys, subjects with one less copy of CNP_8.7 had a very modest 0.01 increase of log eGFRcys (95% CI: -0.02–0.04 ml/min/1.73 m^2^; p = 0.54; n = 6,854). To assess empirically whether the qualitative difference in interpretation of the eGFRcrea and eGFRcys models could be attributable to lack of statistical power in the latter, we re-evaluated the eGFRcrea model using only the 6,854 participants with eGFRcys measurements available. Our findings in the restricted data set are qualitatively similar to the full dataset (effect size = 0.04, p = 0.098).

## Discussion

We implemented a genome-wide association study of CNPs and eGFRcrea in two large EA and AA cohorts represented in ARIC. We identified 312 and 464 CNPs among EA and AA participants, respectively (S1 and S2 Tables). For each CNP, we evaluated copy number in the context of multivariate models for eGFRcrea including known risk factors of kidney disease and principal component-derived surrogates for subpopulation strata in ARIC. While our findings revealed no genome-wide statistically significant associations between copy number and eGFRcrea in either the EA or AA sub-population, we identified one region in the EA cohort close to genome-wide statistical significance (Bonferroni-adjusted p = 0.053). However, we found no evidence to support our expectation that bona fide modulation of latent GFR by copy number dosage would be captured by the alternative GFR surrogate, eGFRcys (p = 0.54). We caution that our secondary analysis using eGFRcys merely provides additional context for the interpretation of the borderline association observed at CNP_8.7 using existing data in ARIC. A definitive analysis of the biological significance of CNP_8.7 (or lack thereof) would require replication in an independent study.

This study extends previous work characterizing copy number variation in ARIC. First, the profile of CNPs in the EA cohort now includes published CNP regions too small for detection by HMMs but estimable by GMMs. Secondly, we provide the first genome-wide profile of CNPs among AA participants in ARIC. In both the EA and AA CNP profiles, the HMM- and HapMap-derived regions were confirmed by GMMs that explicitly model between subject variation of one-dimensional LRR summaries. Finally, we show that 13–16% of the CNPs identified in the EA and AA cohorts would not have been identified by using HapMap information alone. For populations less well-characterized by consortium efforts such as 1000 Genomes and HapMap, these percentages are likely to increase.

In summary, our study does not support a link between CNPs and kidney function as measured by estimated GFR. Nearly 30% of the CNVs identified in this study occur outside of CNPs. The statistical power to detect effect sizes of rare CNVs is limited, particularly in the AA cohort (Figure G in S1 File). Pathway-based analyses and/or meta-analysis of multiple cohorts to study the contribution of rare CNVs in EA and AA subpopulations to CKD require further investigation.

## Methods

### Study population

The ARIC Study is a prospective observational cohort study with participants aged between 45 and 64 at the baseline visit (visit 1) occurring between 1987 and 1989. The participants were recruited from 4 US communities: Forsyth County, North Carolina (F); Jackson, Mississippi (J); suburban Minneapolis, Minnesota (M); and Washington County, Maryland (W). After enrollment, there were three follow-up visits approximately every three years (1990–92, 1993–95, 1996–98). A fifth visit was completed in 2011–2013. Details of the study design have been reported previously[42]. All study participants provided written informed consent, and the study protocol was approved by the Johns Hopkins Bloomberg School of Public Health Institutional Review Board.

### Measurements

In the ARIC study, serum creatinine is available at visits 1, 2, and 4. We used serum creatinine measurements for the visit with the largest participation, visit 1 (n = 15,792). Serum creatinine levels were measured using the modified kinetic Jaffe method and calibrated to the age-, sex-, and race-specific means in the Third National Health and Nutrition Examination Survey (NHANES III). We estimate GFR based on serum creatinine (eGFRcrea) using the Modification of Diet in Renal Disease (MDRD) Study 4-variable equation[10].

Cystatin C, an alternative surrogate quanitative measure of eGFR, was measured by a particle enhanced immunonephelometric assay (N Latex Cystatin C, Dade Behring). The eGFRcys levels were estimated as eGFRcys = 76.7 x (serum cystatin C)^-1.19^ [41]. Both eGFRcrea and eGFRcys were approximately log-normally distributed.

Diabetes was defined as fasting glucose ≥126 mg/dL, non-fasting glucose ≥200 mg/dL, self-reported physician diagnosis of diabetes mellitus or the use of oral hypoglycemic medication or insulin. Hypertension was defined as systolic blood pressure ≥ 140 mmHg, diastolic blood pressure ≥ 90 mmHg or the use hypertension treatment medication.

### Genotyping and quality controls

Genomic DNA was extracted from peripheral whole blood and SNPs were genotyped on the Affymetrix 6.0 chip as described previously[43]. Genetic outliers, first-degree relatives, gender mismatches, and participants who did not consent for use of DNA information were excluded. To control for population stratification, we computed the first 10 principal components using EIGENSTRAT[44] using high quality, independent SNPs. Details of principal component generation have been previously described[45]. All 10 principal components were included as covariates in our statistical model. To summarize, 11,827 participants (9,038 EA and 2,822 AA) attended visit 1, had valid data on serum creatinine, and had genotype data meeting the above quality control criteria (Figure A in S1 File).

### Overview of CNP estimation

To comprehensively identify all CNP regions among EA and AA participants, we pursued a two-step approach. First, we fit a HMM to each individual sample. The HMM allows identification of both CNVs and CNPs. While helpful for identifying CNPs in populations not well represented in public repositories, HMMs have a limited resolution that depends on the inherent marker-to-marker variation of the LRR estimates. As the signal to noise ratio is increased by examining only the markers involved in a CNP across a large collection of samples (e.g., McCarroll et al., 2008[34] and Cardin et al., 2011[37]), we examined the distribution of region-level copy number summaries across all ARIC samples in a second step. In particular, we evaluated all CNPs previously reported in HapMap spanning at least 3 Affymetrix 6.0 markers using a previously described Gaussian mixture model for CNPs[35].

#### Hidden Markov model

B-allele frequencies and wave-adjusted LRRs were computed as described previously[33]. Estimates for copy number states 0–4 for all autosomes were derived as previously described for the EA participants using the VanillaICE HMM[46, 47]. We required at least 10 markers in a CNP region identified by the HMM to reduce false positive identifications. We excluded 393 EA and 275 AA subjects for one or more of the following reasons: median absolute deviation of the LRRs greater than 0.35, autosomal lag 10 autocorrelation of the LRRs greater than 0.05, or more than 150 CNVs (Figure B in S1 File).

#### CNP regions

We refer to CNVs occurring in the population at a frequency of at least 2 percent as CNPs. CNPs tend to have the same or very similar breakpoints across individuals. As CNP regions are known to differ by ancestry, we defined consensus start and stop genomic positions for CNP regions independently for the EA and AA cohorts. Specifically, the consensus start (end) was defined as the minimum (maximum) base-pair spanned by at least half of all CNVs identified by the HMM at a particular polymorphic region. In addition to HMM-derived CNPs, we included 785 candidate CNP regions available from HapMap and reported by McCarroll et al (2008)[34]. For partially overlapping regions, we kept only one region (copy number estimates were nearly identical in each case), yielding 312 non-overlapping regions in EA and 464 non-overlapping regions in AA.

#### Gaussian mixture model

For each HapMap- or HMM-derived CNP candidate, we fit the GMM implemented in the R package cnvCall (Cardin et al., 2011[37]). Briefly, a one-dimensional summary for each sample was derived from the first principal component of the LRR matrix at a CNP (rows are samples and columns are the marker-level LRR). The marginal distribution of the one-dimensional summary (marginal across subjects) was modeled as a mixture of normal distributions. Since the number of mixture components, k, was not known *a priori*, models k = 1 to k = 5 were evaluated at each CNP. The model with the lowest Bayesian Information Criterion (BIC) was then selected. To assign a mixture component index to each sample, we used the maximum *a posteriori* estimate. If the maximum *a posteriori* probability was less than max (0.2, 1/k), an NA, indicating missing, was recorded. A post-post-hoc merging procedure implemented in cnvCall was used to reduce overfitting skewed-normal distributions.

To translate cnvCall component indices into absolute copy number, we implemented a simple relabeling heuristic. Let *I* denote the mixture component index, *I* ∈ {1,…,*k*} and *CN* denote the copy number, *CN* ∈ {0,…,4}. If the average LRR of the first component was less than -1.5 (consistent with a homozygous deletion), we set *CN* = *I*-1. For common deletions and *k* ≥ 3, homozygous deletions often have mean LRR greater than -1.5. Therefore, if the mean of the first component was less than -0.5 and the distance between the first and second mode was 1.5-fold the distance between the second and third mode, we also set *CN* = *I*-1. If the first component is not homozygous (i.e. neither of the above criteria were met), we set *CN* = 2 for the modal component index. The remaining indices were set to *CN* = 1 or *CN* = 3 depending on whether the component index is less or greater than the modal component index, respectively.

From a statistical point of view, two challenging aspects of developing mixture models to estimate copy number are model selection (i.e., choosing the right *k*) and model robustness to assumptions of approximate normality. While cnvCall selects the model with the lowest BIC and has an outlier component to capture outliers, we found that for less common deletions, a model having only hemizygous and diploid components (*k* = 2) was selected over a model that includes a homozygous deletion component (*k* = 3). While the more parsimonious model may be preferable in some situations, here the more parsimonious *k* = 2 model is biologically implausible if the deletion allele is segregating in the population at Hardy Weinberg equilibrium (HWE), as expected. Having implemented the above relabeling heuristic, we identified all CNPs in which the first component was hemizygous deletions. For these CNPs, we set *CN* = 0 for any sample with an average LRR consistent with homozygous deletion (< -1.5) but assigned NA to indicate missing by cnvCall.

#### Analysis of whole genome sequencing platform

We downloaded and preprocessed low-pass whole genome sequencing data for 5 ARIC samples (dbGaP accession number phs000090.v1.p1). Briefly, we realigned each BAM file to the NCBI build 36 reference genome using ELAND[36]. Next, we tiled the genome into 10kb non-overlapping bins and counted the number of reads aligning to each bin. We transformed the bin-counts to the log_2_ scale, and GC-corrected the bin counts using a loess scatterplot smoother with a span of 1/3.

### Association analysis

Copy number was obtained from the relabeled cnvCall component indices and manually identified rare homozygous deletions (as described previously). Other covariates included age, sex, study site, and principal components derived from the SNP genotypes (described previously). Since eGFRcrea is approximately log-normally distributed and previous studies have used the log-transformed response (instead of a log-link), we evaluated linear models with the log-transformed measurements. All CNP analyses were stratified by ancestry. The genome-wide statistical significance level by Bonferroni was p < 1.61x10^-4^ for the EA participants (0.05/312) and p < 1.08x10^-4^ (0.05/464) for the AA participants.

### Statistical power

We used simulation to estimate the statistical power for identifying copy number alterations associated with eGFRcrea levels. Briefly, we randomly sampled the copy number status for 8,645 EA participants assuming prevalence of a deletion allele ranging from rare (0.02, top-left of Figure G in S1) to common (0.2, bottom-right of Figure G in S1). Conditional on the copy number assignment, we added a value β and 2×β to the empirical log(eGFRcrea) estimates for individuals with 1 copy and 2 copies, respectively. We evaluated a range of values for β that include estimates from previously replicated SNP-association studies, as well as values observed in this study. For example, the average slope in a GWAS based on SNPs in the ARIC EA cohort using the same log(eGFRcrea) response was 0.02 [5]. For each simulated dataset, we fit a generalized linear model with log-link and calculated the Bonferroni-adjusted p-value of the copy number regression coefficient. Repeating the simulation 100 times for each combination of deletion prevalence and estimated β, the statistical power is the fraction of simulated datasets with adjusted p < 0.05. We repeated this simulation with a sample size of 2,514 for the AA cohort.

### Genomic annotation and software versions

Genomic annotation in this paper is based on UCSC build hg18 (NCBI36). The version of R and of the R packages used in this analysis is included in S1 File.

## Supporting Information

S1 File Includes Figures A-G and software versions. (DOCX)

S1 Table Genomic coordinates and model summary statistics for EA CNPs. (CSV)

S2 Table Genomic coordinates and model summary statistics for AA CNPs. (CSV)
