# Supplementary material for: Genome-Wide Association of Copy Number Polymorphisms and Kidney Function
Source: PLoS One. 2017 Jan 30;12(1):e0170815. doi: 10.1371/journal.pone.0170815 (PMC5279752; doi:10.1371/journal.pone.0170815)
Supplement: S1 File — (DOCX) [file pone.0170815.s001.docx]

**Genome-wide Association of Copy Number Polymorphisms and Kidney Function**

**Supplementary Material**

Man Li^1,2^, Jacob Carey^1^, Stephen Cristiano^3^, Katalin Susztak^5^, Josef Coresh^1,4^, Eric Boerwinkle^6^, Wen Hong L. Kao^1,4,†^, Terri H. Beaty^1^, Anna Köttgen^1,7^, Robert B. Scharpf^8*^

1. Department of Epidemiology, Johns Hopkins Bloomberg School of Public Health, Baltimore, Maryland, United States of America.
2. Division of Nephrology and Hypertension, Department of Internal Medicine, University of Utah School of Medicine, Salt Lake City, Utah, United States of America.
3. Department of Biostatistics, Johns Hopkins Bloomberg School of Public Health, Baltimore, Maryland, United States of America.
4. Welch Center for Prevention, Epidemiology and Clinical Research, Baltimore, Maryland, United States of America.
5. Renal Electrolyte and Hypertension Division, Perelman School of Medicine, University of Pennsylvania, Philadelphia, Pennsylvania, United States of America.
6. Human Genetics Center, University of Texas Health Science Center at Houston, Houston, Texas, United States of America.
7. Division of Genetic Epidemiology, Medical Center – University of Freiburg, Faculty of, 79106 Freiburg, Germany.
8. Department of Oncology, Johns Hopkins School of Medicine, Baltimore, Maryland, United States of America.

†. Deceased

***** Corresponding author

E-mail: rscharpf@jhu.edu

**Figure A.**  Overview of quality control filters in EA (left) and AA (right) participants.

**Figure B**. To assess the overall quality of the marker-level data derived from the Affymetrix 6.0 platform for copy number analyses, we calculated the median absolute deviation (MAD; y-axis) and the lag 10 autocorrelation (lag10 ACF) of the autosomal log R ratios (LRR). In addition, we calculated the number of autosomal CNVs identified by the hidden Markov model (x-axis). MADs exceeding 0.35 (horizontal dashed line), lag10 ACFs greater than 0.05 (blue points), or number CNVs greater than 150 (vertical dashed line) were excluded from further analysis.

**Figure C. Distribution of CNP locations in AA participants.** Black ticks above the ideograms are additional regions from HapMap identified as polymorphic by the GMM in ARIC. Black ticks below the ideograms are CNPs that are also present in the EA cohort (see also Figure 2).

**Figure D.** Approximately 85 percent of the CNPs in the EA and AA populations have a deletion allele segregating in the population at Hardy Weinberg Equilibrium (HWE p > 0.01). For EA participants, we observed 41 CNPs out of HWE (p < 0.01) compared to 3 expected by chance. CNPs not in HWE reflect the post-hoc merging step (e.g., hemizygous deletion and diploid components were merged for low signal to noise ratio mixtures), mislabeled copy number (e.g., a duplication polymorphism instead of a deletion polymorphism), or an incorrect model selection (number of components in the mixture is incorrect).

**Figure E.** For CNPs identified by the HMM but seen in fewer than 2% of HapMap subjects, we assessed whether the CNP was also rare in other studies. In particular, we examined 17 studies with at least 100 participants in DGV (y-axis). Nearly all of these regions are CNPs (proportion greater than 2%) in one or more of these studies.

**Figure F.** Normalized and GC-adjusted read depth estimates obtained from low-pass whole genome sequencing. Each point corresponds to a 10kb bin. The boxed region is a 44kb CNP on chromosome 5 (8,755,522 – 8,800,142 bp) for which these subjects had a hemizygous deletion identified by the array.

**Figure G. Power calculation.** We used simulation to assess the statistical power for identifying changes in average log(eGFRcrea) per unit change in copy number as a function of the true slope (β) and prevalence of the deletion allele. Here, prevalence is lowest at the top-left and highest at the bottom-right. We expect variants of low prevalence are more likely to have large effects, whereas common deletions (bottom right) may have smaller β.

**Software versions**

We used the May, 2010 version of PennCNV, version 1.14.3 of APT. All remaining analyses were performed in the statistical environment R. Graphics were generated using the R packages ggplot2 and ggbio. The complete listing of supporting R packages and their corresponding version numbers is provided below.
